# Atypical cause of intractable diarrhea in a hemodialysis patient, masked by *Clostridium difficile*-associated diarrhea and ischemic colitis: a case report

**DOI:** 10.1186/s12882-018-1116-x

**Published:** 2018-11-01

**Authors:** Takaaki Higashihara, Akira Okada, Yukiko Kishida, Sayako Maruno, Mimiko Matsumura, Koichi Tamura, Hideki Takano

**Affiliations:** 1 Department of Nephrology, Tokyo Teishin Hospital 2-14-23, Fujimi, Chiyoda-ku, Tokyo, 102-8798 Japan; 2 Division of Nephrology and Endocrinology, The University of Tokyo Graduate School of Medicine, 7-3-1, Hongo, Bunkyo-ku, Tokyo, 113-8655 Japan; 3 Department of Pathology, Tokyo Teishin Hospital 2-14-23, Fujimi, Chiyoda-ku, Tokyo, 102-8798 Japan

**Keywords:** Diarrhea, Ulcerative colitis, *Clostridium difficile* associated diarrhea, Ischemic colitis, Hemodialysis, Immune dysfunction, Case report

## Abstract

**Background:**

Patients with end-stage kidney disease (ESKD) most commonly complain of gastrointestinal symptoms, such as diarrhea. Diarrhea negatively affects patient quality of life and has miscellaneous etiologies, such as *Clostridium difficile-*associated diarrhea (CDAD) and ischemic colitis. However, it is sometimes extremely difficult to determine the true etiology given the comorbidities and complications the patients have. A rare cause of diarrhea is ulcerative colitis (UC), which commonly affects the rectum and proximal colon in a continuous fashion. UC with rectal sparing or segmental distribution, although atypical, sometimes leads to misdiagnosis. Herein, we present a case of UC in a patient on hemodialysis with intractable diarrhea; we initially considered that the diarrhea was caused by CDAD and ischemic colitis.

**Case presentation:**

A 69-year-old man with a history of hypertension, bilateral thalamic hemorrhage, and decreased kidney function was admitted to our hospital because of congestive heart failure. Volume control was impossible due to renal dysfunction and he was started on hemodialysis. Thereafter, he received various antibiotics for bacterial infections. Simultaneously, he experienced continuous watery, and sometimes bloody, diarrhea, which was diagnosed as CDAD owing to a positive stool test for *Clostridium difficile* toxins. Antibiotic treatment for CDAD did not result in symptom relief. Subsequently, we performed colon biopsy via colonoscopy, and the pathology showed virtually no inflammation with rectal sparing and segmental distributions. These findings favored the presence of ischemic colitis due to arteriosclerosis and ESKD rather than infections. He died of cardiac arrest before the diarrhea was alleviated. Finally, UC was revealed on autopsy as the main cause of the uncontrollable diarrhea.

**Conclusions:**

Patients with ESKD have a greater risk of developing CDAD and ischemic colitis, which have clinical features that sometimes overlap with those of UC, as in the present case. This case emphasizes the importance of correctly diagnosing the etiology of intractable diarrhea and the fact that other diarrhea etiologies can obscure the existence of inflammatory bowel disease, which should be considered and treated properly when patients on hemodialysis present with intractable diarrhea.

## Background

Patients with end-stage kidney disease (ESKD) often have gastrointestinal presentations, including diarrhea, which has a variety of etiologies [1, 2]. Patients with ESKD have a greater risk of *Clostridium difficile-*associated diarrhea (CDAD) [3, 4] and ischemic colitis [5], which are reportedly associated with immune dysfunction [6–8] and atherosclerotic cardiovascular disease, respectively [9]. One of the rare causes of diarrhea is ulcerative colitis (UC), which is an idiopathic, chronic inflammatory disorder of the colonic mucosa [10]. UC generally involves the rectum and proximal colon with a continuous and non-segmental distribution and is characterized by biopsy features such as cryptitis with crypt abscesses [11, 12]. However, these findings are not always specific to UC [13] and atypical cases with rectal sparing or segmental distribution have been reported [14]. Moreover, the clinical features of UC sometimes overlap with other relatively common causes of diarrhea, such as CDAD and ischemic colitis [15, 16]. Therefore, UC with atypical features can be extremely difficult to diagnose correctly in a patient with ESKD. Herein, we present a rare case of a patient with ESKD, in whom UC was obscured by overlap with CDAD and suspected ischemic colitis.

## Case presentation

A 69-year-old man with a history of hypertension, bilateral thalamic hemorrhage, and decreased kidney function was admitted to our hospital because of congestive heart failure with extracellular volume overload. Volume control with drugs, such as diuretics, was unsuccessful and he was started on hemodialysis. Thereafter, he received various antibiotics for bacterial infections, including pneumoniae and urinary-tract and catheter-related infections. Simultaneously, he had continuous watery and sometimes bloody diarrhea, the etiology of which was considered to be CDAD due to a positive stool test for CD toxins. Despite treatment with metronidazole and vancomycin, the severity of the diarrhea was not ameliorated. Abdominal X-ray and computed tomography did not reveal any causal factors, and laboratory tests showed only slight elevations in the white blood cell count and C-reactive protein level. We suspected involvement of uncontrollable CDAD or other type of infection (such as mycosis, tuberculosis, or cytomegalovirus infection) and inflammatory bowel disease (IBD).

**Fig. 1:**
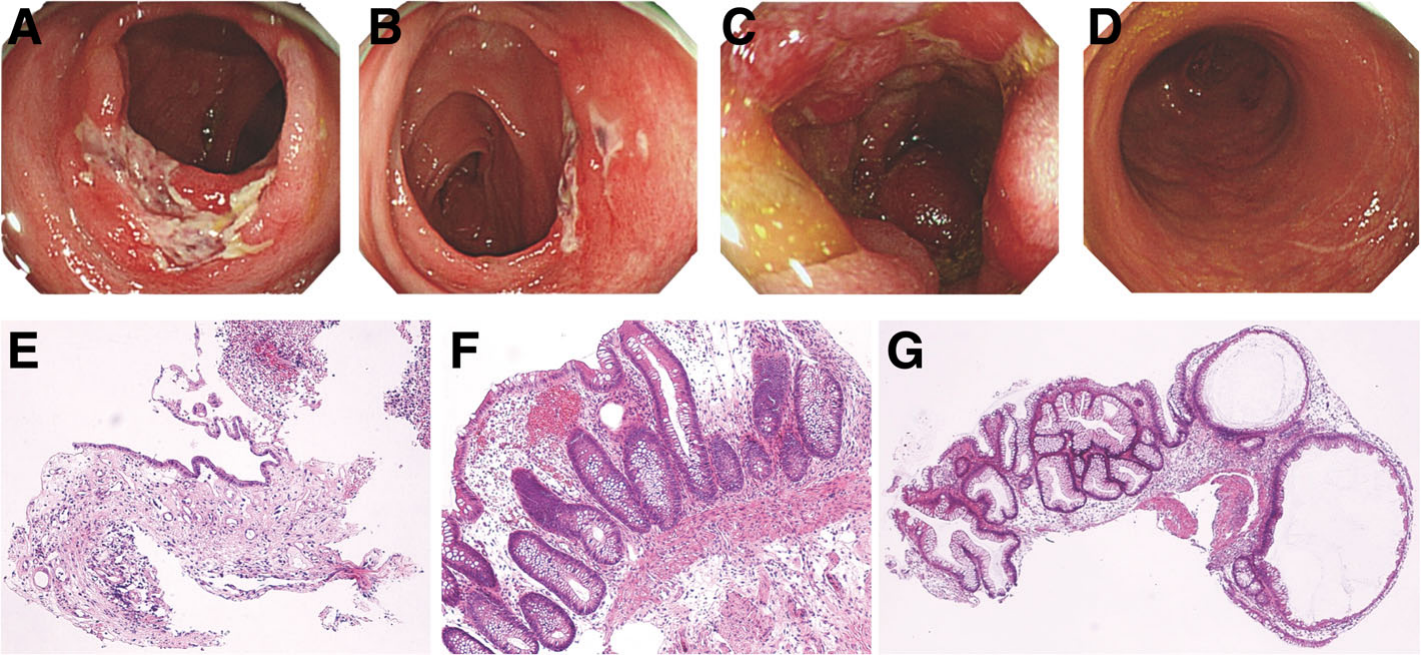
Colonoscopic findings and light microscopic images on the initial colon biopsy. Redness, erosions, and segmental ulcers are observed in the ascending colon (**a**), transverse colon (**b**), and sigmoid colon (**c**). In contrast, there are no rectal involvements (**d**). Light microscopic images show loss of glands and fibrosis in the mucosa of the ascending colon (**e**) and congestion, edema, and partial loss of glands in the transverse colon (**f**); these findings are compatible with ischemic colitis. Hyperplastic glands with dilatation can be observed in the sigmoid colon, with no other specific findings such as infection (**g**)

**Fig. 2:**
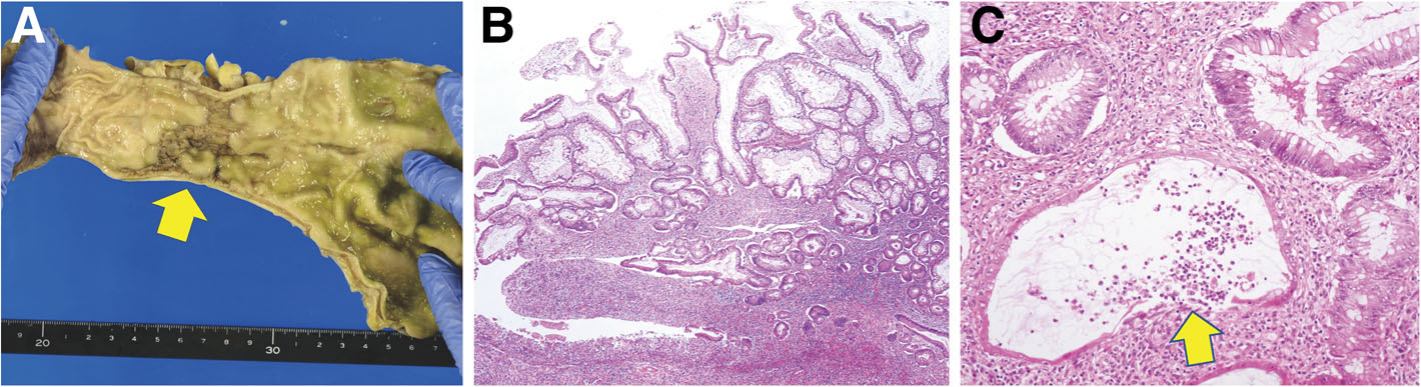
Macroscopic and light microscopic images on autopsy. Longitudinal and annular ulcers are observed in the sigmoid-colon (*arrows*) (**a**). Light microscopy shows hyperplastic foveolar epithelium around the ulcers, with lymphocytic aggregates near the muscularis mucosa (**b**) and cryptitis with crypt abscess formation (*arrows*) (**c**)

Colonoscopy showed multiple segmental ulcers in the ascending, transverse, and sigmoid colon, but not the rectum, which suggested infectious colitis or ischemic colitis (Fig. 1). A biopsy specimen showed loss of glands, fibrosis, congestion, and edema, suggesting ischemia, with no findings of infection (Fig. 1). Given these results and the fact that the patient was on dialysis and had severe arteriosclerosis, we provisionally diagnosed ischemic colitis caused by arteriosclerosis and hemodialysis. We stopped or adjusted enteral nutrition, avoided low blood pressure, and withdrew antibiotics; however, his diarrhea persisted. It was impractical to perform a second colonoscopy, as his performance status was severely worsened. Finally, he died of cardiac arrest 193 days after admission. The autopsy showed longitudinal and annular ulcers in the sigmoid colon. An autopsy specimen of the ulcers showed inflammatory polyposis, cryptitis with crypt abscess formation, and focally severe lymphocyte infiltration near the muscularis mucosa; these findings are fully compatible with UC (Fig. 2). We retrospectively suspected that the cause of the intractable diarrhea was UC, which had been masked by the clinical course and findings of CDAD and ischemic colitis.

## Discussion and conclusions

Chronic kidney disease is characterized by uremia-related immune disorders of both the innate and adaptive systems, resulting in an increased risk of cardiovascular and infectious diseases [17]. Patients on hemodialysis are thus considered to be a compromised population and are likely to develop several infections requiring antibiotic treatment. Thus, such patients also have an increased risk of antibiotic-associated diarrhea, such as CDAD [3, 4]. Consistent with this, our patient recurrently received antibiotics and his case was complicated with CDAD, which was diagnosed based on the clinical history and a stool test for CD toxins [18]. However, infectious colitis, including CDAD, is sometimes difficult to distinguish from an acute onset or relapse of UC due to the similarity in clinical presentations and false positives/negatives on a CD toxin test [4, 12, 15]. Endoscopy and colon biopsies are desirable for a correct diagnosis. We succeeded in excluding at least uncontrolled CDAD on the first colon biopsy, even though CDAD might have continued to affect the gastrointestinal tract of the patient. However, it was difficult to judge whether the recurrent CDAD affected the intractable diarrhea or not, as antibiotics were frequently used after the first biopsy.

Furthermore, ESKD increases the risk for ischemic colitis [5]. Reportedly, endoscopic findings of a segmental distribution with rectal sparing can distinguish between UC and ischemic colitis [19]. However, past studies have reported that 19.2% of patients with UC show an atypical distribution at the initial colonoscopy and 3.3% of patients have rectal sparing (segmental-type UC) [14]. Moreover, ischemic colitis and UC in elderly individuals (age > 60 years) are said to have similarities in clinical characteristics [16]. In the present case, the first colonoscopy and biopsy were suggestive of ischemic colitis, rather than UC. However, it is possible that UC was also present at the time of the first biopsy. Continuous intractable diarrhea after the diagnosis of ischemic colitis implied the involvement of other causes. However, a definitive diagnosis of UC was extremely difficult, as there were no extra-intestinal manifestations of UC, and tetraplegia and dysarthria due to bilateral thalamic hemorrhage hindered detailed physical examinations and a second colonoscopy. The autopsy showed colon ulcers in only the sigmoid colon, with histologic findings fully compatible with UC. Other ulcers in the transverse and ascending colon previously observed had vanished probably because the ischemic colitis could have resolved with the passage of time. Some of the segmental ulcers may have been caused by ischemic colitis, which was complicated with UC. In summary, the present case is enlightening to physicians regarding the importance of correctly diagnosing the etiology of intractable diarrhea because a correct diagnosis can improve the prognosis. At the same time, this case highlights the fact that it is challenging to diagnose UC with atypical features in a patient on hemodialysis, as the clinical features of CDAD and ischemic colitis sometimes overlap with those of UC. Therefore, the possibility of IBD, including UC, should be considered in cases of intractable diarrhea in patients on hemodialysis if detailed evaluation is possible. More cases similar to the present one should be reported and accumulated to better understand the differential diagnoses and management of diarrhea in patients with ESKD.

## Abbreviations

CDAD: *Clostridium difficile*-associated diarrhea
ESKD: End-stage kidney disease
IBD: Inflammatory bowel disease
UC: Ulcerative colitis
